# Vitamin B12 deficiency in metformin-treated adults with type 2 diabetes mellitus in Africa: A systematic review and meta-analysis

**DOI:** 10.1016/j.metop.2026.100524

**Published:** 2026-11-02

**Authors:** Kibrom Aregawi Amare, Niguse Shishay Kahsay, Bahlbi Hailay Abraha, Yemane Mengsteab Hagos, Merhawi Alemu Birhanu, Abel Deres Teka, Mebrahtu Tefera Aynalem

**Affiliations:** Department of Medical Laboratory Science, College of Medicine and Health Sciences, Adigrat University, Adigrat, Tigray, Ethiopia

**Keywords:** Vitamin B12 deficiency, Metformin, Type 2 diabetes mellitus, Africa, Meta-analysis

## Abstract

**Background:**

Vitamin B12 deficiency is a clinically important complication of long-term metformin therapy and may contribute to hematological and neurological complications in patients with type 2 diabetes mellitus. Although metformin is widely used in Africa, comprehensive evidence on the prevalence and associated factors of vitamin B12 deficiency among metformin-treated patients remains limited and fragmented. This systematic review and meta-analysis aimed to estimate the pooled prevalence of vitamin B12 deficiency and identify factors associated with its occurrence among adults with type 2 diabetes mellitus receiving metformin in Africa.

**Methods:**

Following PRISMA 2020 guidelines, we searched PubMed, Cochrane Library, AJOL, AIM, DOAJ, ScienceDirect, Google Scholar, and reference lists from January 1, 2000, to October 18, 2026. Eligible studies reported the prevalence of vitamin B12 deficiency in metformin-treated adults with type 2 diabetes mellitus. Methodological quality was assessed using JBI tools, and pooled prevalence was estimated using a random-effects REML model in Stata 17.0.

**Results:**

Nineteen studies involving 3108 adults with type 2 diabetes mellitus from 10 African countries were included. The pooled prevalence of vitamin B12 deficiency was 14.7% (95% CI: 9.7–21.6%), with substantial between-study heterogeneity (I^2^ = 94.69%) and a 95% prediction interval of 2.0–59.7%, indicating considerable variation in the expected prevalence across different clinical and population settings. The included studies used heterogeneous study-defined diagnostic criteria and laboratory methods for vitamin B12 deficiency. Therefore, the pooled estimate should be interpreted as a summary of the available African studies rather than as a continent-wide prevalence estimate. Narrative synthesis suggested that older age, longer metformin exposure, and higher metformin doses were the most consistently reported factors associated with vitamin B12 deficiency.

**Conclusion:**

The available African studies yielded a pooled prevalence of approximately 14.7%, based on study-specific definitions of vitamin B12 deficiency. Vitamin B12 assessment may be particularly relevant in patients receiving long-term or high-dose metformin, especially when neurological or hematological manifestations are present.

**Systematic review registrations:**

PROSPERO (CRD420261463974).

## 1 Background

Type 2 diabetes mellitus (T2DM) is a major and growing global health problem that requires sustained management to achieve glycemic control and reduce the risk of long-term complications [1]. Metformin is widely used as a first-line oral glucose-lowering therapy because of its effectiveness, affordability, low risk of hypoglycemia, and established safety profile [2], [3]. However, prolonged metformin use and higher doses have been associated with an increased risk of vitamin B12 deficiency [2], [4].

Vitamin B12 is essential for DNA synthesis, myelin formation, erythropoiesis, and neurological function [5]. Metformin use has been associated with reduced circulating vitamin B12 concentrations, and evidence from systematic reviews and meta-analyses indicates that metformin-treated individuals with T2DM have a higher risk of vitamin B12 deficiency than those not receiving metformin [6]. Although the underlying mechanisms are not completely understood, impaired intestinal absorption of vitamin B12 is considered an important pathway.

Importantly, vitamin B12 status can be assessed using different biochemical approaches that do not necessarily identify the same biological state. Total serum vitamin B12 concentration is commonly used to identify biochemical vitamin B12 deficiency based on a predefined concentration threshold, whereas metabolic biomarkers such as methylmalonic acid (MMA) can provide complementary information on the functional consequences of inadequate intracellular vitamin B12 availability [7]. Composite approaches that combine multiple biomarkers, including total vitamin B12, holotranscobalamin, MMA, and homocysteine, have also been developed to provide an integrated assessment of vitamin B12 status [8]. These approaches may identify different aspects of vitamin B12 insufficiency and can therefore produce different estimates of deficiency within the same population. Consequently, variation in diagnostic criteria, biomarker selection, and laboratory methods across studies may contribute to between-study heterogeneity in estimates of vitamin B12 deficiency [7], [8].

One proposed mechanism involves interference with the calcium-dependent uptake of the intrinsic factor–vitamin B12 complex by the cubilin receptor in the terminal ileum, thereby reducing vitamin B12 absorption. Other proposed mechanisms include altered gastrointestinal motility and bacterial overgrowth, reduced intrinsic factor secretion, changes in hepatic vitamin B12 uptake or metabolism, and alterations in bile acid metabolism and the enterohepatic circulation of vitamin B12 [9].

The risk of vitamin B12 deficiency among metformin-treated adults with T2DM appears to increase with higher metformin doses and longer treatment duration. Other factors that may contribute to vitamin B12 deficiency include older age, inadequate dietary intake, gastrointestinal disorders that impair vitamin B12 absorption, and concomitant use of acid-suppressive medications, including proton pump inhibitors and histamine-2 receptor antagonists. However, evidence for these factors is less consistent than that for metformin dose and treatment duration and may vary across populations and clinical settings [9], [10].

The clinical importance of vitamin B12 deficiency in T2DM extends beyond its hematological manifestations. Vitamin B12 deficiency can cause neurological abnormalities, including peripheral sensory symptoms, that may overlap with manifestations of diabetic peripheral neuropathy [11]. Because diabetic peripheral neuropathy is a common complication of T2DM, distinguishing its manifestations from other potentially treatable causes of peripheral neuropathy, including vitamin B12 deficiency, can be clinically challenging [12].

The burden of diabetes is increasing across Africa alongside demographic, nutritional, and lifestyle transitions, including urbanization, population aging, increasing obesity, and changing dietary patterns [13]. The growing number of adults with T2DM receiving long-term metformin therapy therefore underscores the potential importance of vitamin B12 deficiency in African diabetes care [9]. Additional challenges to detecting vitamin B12 deficiency in African settings may include delayed diabetes diagnosis, nutritional insecurity, dietary patterns, limited routine screening, and restricted access to vitamin B12 testing [14], [15], [16].

Although previous systematic reviews and meta-analyses have examined the relationship between metformin use and vitamin B12 deficiency among people with T2DM internationally [17], [18], [19], [20], these syntheses have largely combined evidence from diverse geographical settings and have not specifically characterized the African evidence base. Evidence from African populations remains geographically fragmented, and, to our knowledge, no previous systematic review and meta-analysis has comprehensively synthesized the prevalence and factors associated with vitamin B12 deficiency among metformin-treated adults with T2DM across Africa. A continent-focused synthesis is important because estimates derived predominantly from non-African populations may not adequately reflect the epidemiological, nutritional, healthcare, and diagnostic contexts of African settings. Furthermore, characterizing between-study variability and factors associated with vitamin B12 deficiency may help inform risk assessment, clinical monitoring, and future research priorities.

Therefore, this systematic review and meta-analysis aimed to estimate the pooled prevalence of vitamin B12 deficiency among adults with T2DM receiving metformin in Africa, identify factors reported to be associated with vitamin B12 deficiency, and explore variation in prevalence across geographical and methodological settings. By synthesizing the available African evidence, this study seeks to provide clinically relevant estimates, characterize factors associated with vitamin B12 deficiency, and identify evidence gaps that may inform clinical monitoring and future research.

## 2 Materials and methods

### 2.1 Protocol and registration

This systematic review and meta-analysis was conducted and reported according to the Preferred Reporting Items for Systematic Reviews and Meta-Analyses (PRISMA 2020) guidelines [21]. The completed PRISMA 2020 checklist is provided in Supplementary File (SF1). The study selection process was documented using a PRISMA 2020 flow diagram illustrating the number of records identified, screened, assessed for eligibility, and included in the final synthesis (Fig. 1). Records retrieved from the seven electronic search sources, including Google Scholar, were checked for duplicates before screening. Records identified through citation searching and reference-list screening were subsequently cross-checked against the database search results and against each other to identify and eliminate overlapping records. The review protocol was registered in the International Prospective Register of Systematic Reviews (PROSPERO) to promote transparency, reduce unnecessary duplication, and minimize selective reporting bias. The registration details, including the review objectives, eligibility criteria, search strategy, and planned methods for data synthesis, were made publicly available.

**Fig. 1:**
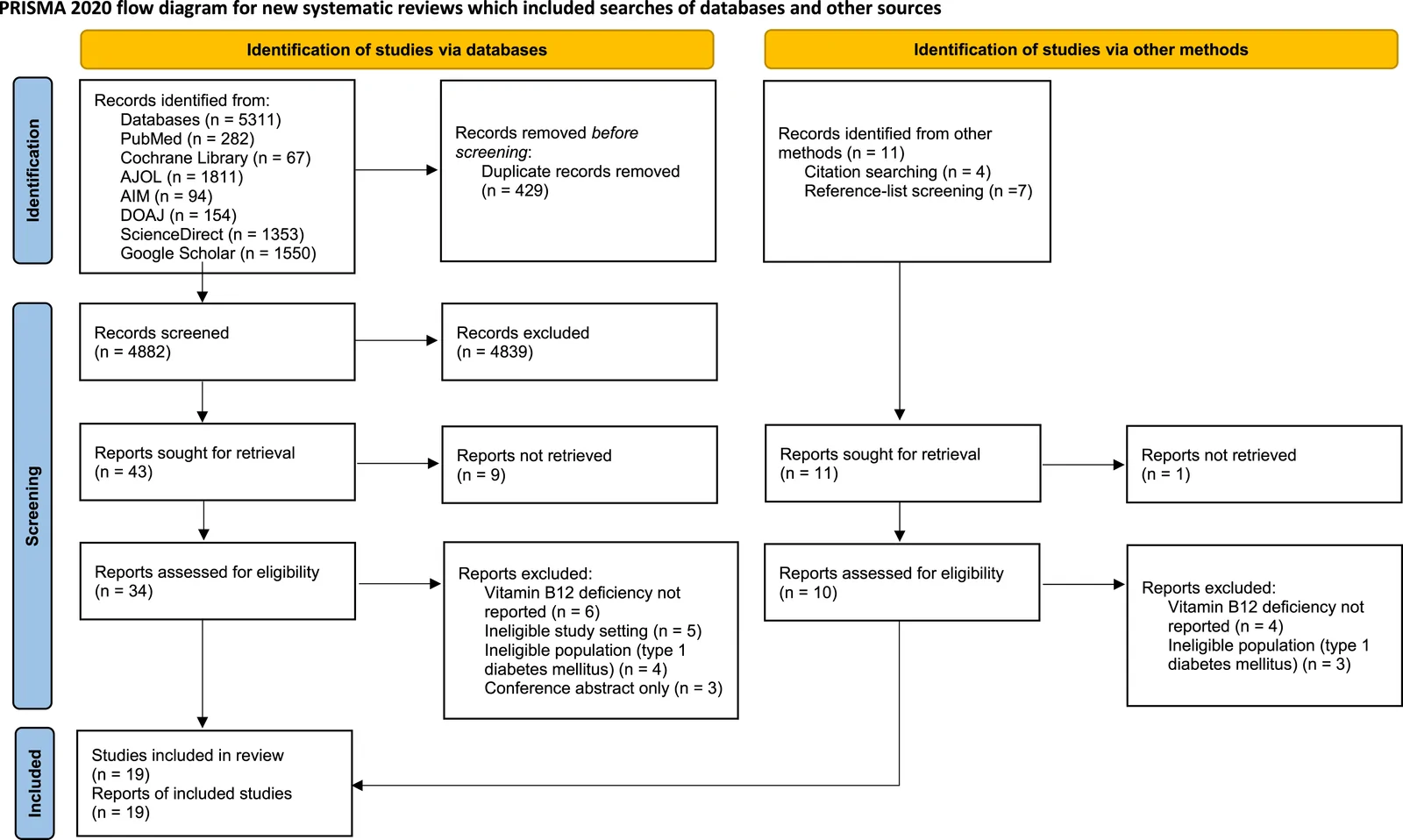
PRISMA 2020 flow diagram of the study selection process for the systematic review and meta-analysis of vitamin B12 deficiency in metformin-treated adults with type 2 diabetes mellitus in Africa. Note: AJOL, African Journals Online; AIM, African Index Medicine; DOAJ, Directory of Open Access Journals.

### 2.2 Eligibility criteria

Studies were eligible for inclusion if they were conducted in an African country and involved adults (≥18 years) with T2DM receiving metformin therapy. Eligible studies were required to report information on vitamin B12 deficiency prevalence and/or factors associated with vitamin B12 deficiency. We considered studies reporting vitamin B12 status assessed using serum vitamin B12 alone or in combination with other biochemical biomarkers, provided that sufficient information was available to determine the study-defined vitamin B12 deficiency status.

For the quantitative prevalence meta-analysis, studies were required to provide a prevalence estimate of vitamin B12 deficiency or sufficient numerical information to calculate the prevalence, including the number of participants classified as deficient and the corresponding denominator among metformin-treated participants. Only prevalence estimates that could be clearly attributed to the metformin-treated population were included in the quantitative synthesis.

For the narrative synthesis of associated factors, studies were eligible if they reported information on factors associated with vitamin B12 deficiency among metformin-treated adults with T2DM, even when they did not provide sufficient data for inclusion in the quantitative prevalence meta-analysis. Reported factors were synthesized narratively when quantitative pooling was not appropriate because of differences in exposure definitions, reference categories, statistical measures, or adjustment methods.

Studies were excluded if they focused exclusively on type 1 diabetes mellitus, gestational diabetes, or other forms of diabetes without separately reporting data for adults with T2DM; included participants younger than 18 years; did not involve metformin therapy; or did not provide relevant information on vitamin B12 deficiency or its associated factors. Reviews, meta-analyses, case reports, case series, conference abstracts, editorials, letters, commentaries, protocols, and other non-original publications were also excluded. Duplicate publications and studies with insufficient information to determine eligibility or extract relevant data were excluded.

### 2.3 Study design and search strategy

This systematic review and meta-analysis included observational studies conducted in African countries that evaluated vitamin B12 deficiency among adults with T2DM receiving metformin therapy. Although two included studies were described by their authors as case-control studies, their vitamin B12 prevalence estimates were calculated within the metformin-treated groups rather than from participants selected according to vitamin B12 deficiency status. These studies were therefore retained in the primary prevalence synthesis, while their potential influence was examined through pre-specified sensitivity analyses excluding case-control studies and restricting the analysis to cross-sectional studies only.

A comprehensive literature search was conducted in PubMed/MEDLINE, the Cochrane Library, African Journals Online (AJOL), African Index Medicus (AIM), the Directory of Open Access Journals (DOAJ), ScienceDirect, and Google Scholar. Additional studies were identified through citation searching and screening the reference lists of relevant articles.

Database-specific search strategies were developed using controlled vocabulary and free-text terms related to vitamin B12 deficiency, metformin, and T2DM, with adaptations made to the indexing requirements of each information source. The complete search strategies, search dates, platforms, applied limits, and numbers of records retrieved from each source are provided in Supplementary File (SF2).

### 2.4 Variable definition and measurement

The primary outcome of this systematic review and meta-analysis was the prevalence of vitamin B12 deficiency among metformin-treated adults with T2DM in Africa. Vitamin B12 deficiency was defined according to the diagnostic criteria reported by each included study. Because diagnostic definitions varied across studies, including serum vitamin B12 concentration thresholds (e.g., ≤200 pmol/L, <150 pmol/L, and <200 pg/mL), as well as definitions based on additional biochemical biomarkers, the specific definition and corresponding cutoff value used by each study were extracted and documented.

Vitamin B12 status was assessed using laboratory-based methods reported by the individual studies, including high-performance liquid chromatography (HPLC), chemiluminescent immunoassay (CLIA), electrochemiluminescence immunoassay (ECLIA), and enzyme-linked immunosorbent assay (ELISA). Where reported, additional biomarkers and composite measures of vitamin B12 status, including MMA and the combined indicator of vitamin B12 status (4cB12) were also extracted. These approaches are not necessarily interchangeable, as they may capture different aspects of vitamin B12 status and may classify vitamin B12 deficiency differently.

Given the absence of a single standardized diagnostic definition across the included studies, the primary meta-analysis was interpreted as a synthesis of study-defined vitamin B12 deficiency rather than as an estimate based on a uniform diagnostic threshold. The variation in diagnostic definitions and laboratory methods was considered an important source of clinical and methodological heterogeneity. Accordingly, definitions, cutoff values, and laboratory measurement methods were extracted using a standardized data extraction form and were considered when interpreting between-study heterogeneity and conducting subgroup or sensitivity analyses. Where a sufficient number of studies used comparable serum vitamin B12 thresholds, a sensitivity analysis restricted to these studies was performed to assess the robustness of the primary pooled estimate.

### 2.5 Study selection and quality assessment

All records identified through the database searches were imported into EndNote version 20, and duplicate records were removed. The titles and abstracts of the retrieved records were screened against the predefined eligibility criteria by two independent reviewers (KA and NS). Full-text articles of potentially eligible studies were then retrieved and independently assessed for final inclusion. During full-text assessment, potentially overlapping populations or multiple publications from the same study setting were assessed using study setting, recruitment period, sample size, participant characteristics, authorship, and other study identifiers. Where overlapping populations were identified, only the most complete and relevant report was retained, and reports without independent participant data were not counted separately to avoid double-counting. Disagreements regarding eligibility were resolved through discussion between the reviewers. If consensus could not be reached, a third reviewer (YM) arbitrated. A PRISMA flow diagram was used to summarize the study selection process, including the numbers of records identified, screened, excluded, assessed for eligibility, and included in the qualitative and quantitative syntheses, with reasons for full-text exclusions where applicable.

Two reviewers (KA and AD) independently assessed the methodological quality and risk of bias of the included studies using the Joanna Briggs Institute (JBI) critical appraisal tools appropriate to each study design [22]. The JBI Critical Appraisal Checklist for Studies Reporting Prevalence Data was used for studies contributing prevalence estimates [23], while case-control studies were assessed using the JBI Critical Appraisal Checklist for Case-Control Studies [22]. For studies described as retrospective, the underlying study design was determined from the methods, and the corresponding JBI appraisal tool was applied. Appraisal was based primarily on the individual checklist items and methodological domains. The detailed item-level judgments are presented in Supplementary Table (SF3).

### 2.6 Data extraction

Data were extracted independently by two reviewers (KA and BH) using a standardized Microsoft Excel spreadsheet. Discrepancies were resolved through discussion with a third reviewer (MA). The data extraction form was used to collect information on author name, publication year, study country and geographical region, study design, sample size, definitions and cutoff values used to diagnose vitamin B12 deficiency, methods used to assess vitamin B12 status, prevalence of vitamin B12 deficiency, and reported factors associated with vitamin B12 deficiency (SF4).

### 2.7 Statistical analysis

The extracted data were entered and analyzed using Stata version 17.0. Pooled prevalence was estimated using a random-effects meta-analysis with restricted maximum likelihood (REML) based on logit-transformed prevalence estimates; pooled estimates and 95% confidence intervals (CIs) were back-transformed to the prevalence scale. Pooling was considered appropriate because all included studies estimated the proportion of metformin-treated adults with T2DM classified as vitamin B12 deficient according to study-defined diagnostic criteria, while differences in thresholds and laboratory methods were treated as sources of clinical and methodological heterogeneity rather than automatic reasons for exclusion. A random-effects model was used because variation in prevalence was expected across study populations, geographic settings, diagnostic criteria, and laboratory methods. Statistical heterogeneity was assessed using Cochran's Q, τ^2^, I^2^ statistics, and Galbraith plots; I^2^ values of 25%, 50%, and 75% indicated low, moderate, and high heterogeneity, respectively [24]. A 95% prediction interval (PI) was calculated to estimate the range within which the true prevalence of a future comparable study would be expected to lie.

Subgroup analyses and univariate meta-regression were performed as exploratory analyses to examine whether selected study-level characteristics were associated with variation in the pooled prevalence and the observed between-study heterogeneity. Sensitivity analyses were conducted to assess the robustness of the pooled prevalence estimate to differences in study design and diagnostic definitions. Regarding study design, the meta-analysis was repeated after excluding the two studies classified by their authors as case-control studies and was further restricted to cross-sectional studies. To evaluate the influence of diagnostic heterogeneity, an additional sensitivity analysis was restricted to studies using approximately comparable serum vitamin B12 deficiency thresholds centered on 200 pg/mL. Reported thresholds were standardized to pg/mL using the conversion formula pg/mL = pmol/L ÷ 0.7378, while values expressed in pg/dL were divided by 100. An operational equivalence range of 197.00–203.31 pg/mL (approximately 145.35–150 pmol/L) was established post hoc to accommodate minor differences in reported thresholds. Studies using substantially different serum vitamin B12 thresholds, combined or alternative diagnostic indicators (MMA or 4cB12), or unreported thresholds were excluded. The study reporting a cutoff of <200 pg/dL was also excluded because its reported unit corresponds to 2 pg/mL rather than 200 pg/mL. Study-specific thresholds, standardized values, and inclusion decisions are detailed in Supplementary Table (SF5).The same logit transformation and REML random-effects model used in the primary analysis were applied to all sensitivity analyses.

Potential small-study effects were explored using funnel-plot asymmetry, Egger's test, and Begg's rank correlation test.

## 3 Results

### 3.1 Search results and included studies

The literature search identified 5322 records, comprising 5311 from database searches and 11 through other methods, covering publications from 1 January 2000 to 18 October 2026. Following the removal of 429 duplicate records across all seven database sources, including Google Scholar, 4882 records were screened by title and abstract, of which 4839 were excluded. Subsequently, 43 reports were sought for retrieval, but nine could not be retrieved, leaving 34 reports for full-text eligibility assessment. Of these, 18 reports were excluded because vitamin B12 deficiency was not reported (n = 6), the study setting was ineligible (n = 5), the study population was ineligible because it included patients with type 1 diabetes mellitus (n = 4), or the publication was available only as a conference abstract (n = 3).

An additional 11 records were identified through other methods, including citation searching (n = 4) and reference-list screening (n = 7). These records were cross-checked against the database search results and against each other, and no duplicates were identified. Of these, one report could not be retrieved, leaving 10 reports for eligibility assessment. Seven reports were subsequently excluded because of an ineligible study population involving patients with type 1 diabetes mellitus (n = 3) or the absence of reported vitamin B12 deficiency outcomes (n = 4).

Overall, 19 reports representing 19 studies met the eligibility criteria and were included in the systematic review and meta-analysis (Fig. 1).

### 3.2 Characteristics of the included studies

A total of 19 studies involving 3108 participants from 10 African countries were included in the systematic review and meta-analysis. Fourteen studies were cross-sectional, while three were retrospective and two were case-control studies. Among the 18 studies that reported sex distribution, approximately 1903 (62.9%) participants were female and 1124 (37.1%) were male. One study involving 81 participants did not report the sex distribution [25].

Two studies were described by their authors as case-control studies. In Owhin et al., 100 metformin-treated participants contributed to the prevalence estimate, although the overall study included 200 participants, comprising 100 metformin-treated and 100 metformin-naïve participants. In Mohammed and Abd Elkarim, 50 metformin-treated participants contributed to the prevalence estimate from an overall study population of 100 participants. In both studies, participants were classified according to metformin exposure, and vitamin B12 status was assessed subsequently rather than participants being selected according to vitamin B12 deficiency status. Accordingly, the prevalence denominators used in the meta-analysis were the metformin-treated groups (100 and 50 participants, respectively), rather than the total study populations.

Sample sizes ranged from 30 participants in Egypt [26] to 500 participants in Libya [27]. The included studies were conducted across 10 African countries: Egypt (three studies) [26], [28], [29]; South Africa (three studies) [30], [31], [32], Nigeria (two studies) [25], [33], Ghana (two studies) [34], [35], Libya (four studies) [27], [36], [37], [38]; and Ethiopia [39], Tunisia [40], Uganda [41], Tanzania [42], and Sudan [43], with one study from each country.

Across the included studies, a total of 471 cases of vitamin B12 deficiency were reported. Most studies assessed vitamin B12 deficiency using serum vitamin B12 concentrations. One study assessed vitamin B12 status using serum vitamin B12 in combination with MMA [35], while another used the combined indicator of vitamin B12 status (4cB12) [34]. Laboratory measurements were performed using CLIA, ECLIA, ELISA, and HPLC, with varying diagnostic cutoff values across studies. The overall mean age of participants was 56.93 years (Table 1).

**Table 1:** Characteristics of the included studies on vitamin B12 deficiency among metformin-treated adults with type 2 diabetes mellitus in Africa.

Author name	Country	Region	Publication year	Study design	Sample size	Mean Age	Male (%)	Female (%)	Cutoff value	Vitamin B12 assessment measure	Laboratory assay	Number of cases
Ahmed MA et al. [30]	South Africa	Southern Africa	2016	cross-sectional	121	58	44.6	55.4	<150 pmol/L	Serum vitamin B12	CLIA	34
Sakyi, S. A. et al. [34]	Ghana	West Africa	2021	cross-sectional	200	59	29	71	4cB12 (ω < −0.5)	4cB_12_	ECLIA + ELISA	81
Owhin, S. O. et al. [33]	Nigeria	West Africa	2019	Case-control	100	55.8	49	51	<148 pmol/L	Serum vitamin B12	ELISA	41
Akinlade, K. S. et al. [25]	Nigeria	West Africa	2015	cross-sectional	81	61.96	NR	NR	<200 pg/dL	Serum vitamin B12	HPLC	7
Wale Tesega, W. et al. [39]	Ethiopia	East Africa	2021	cross-sectional	80	56.35	42.5	57.5	<197 pg/mL	Serum vitamin B12	CLIA	4
Kondlo, B. et al. [31]	South Africa	Southern Africa	2024	Retrospective	101	70.2	35	65	≤200 pmol/L	Serum vitamin B12	ECLIA	15
Rojbi, I. et al. [40]	Tunisia	North Africa	2023	cross-sectional	120	59	49.2	50.8	<200 pg/mL	Serum vitamin B12	NR	8
Ali, H. O. et al. [41]	Uganda	East Africa	2026	cross-sectional	257	56.3	51	49	<150 pg/mL	Serum vitamin B12	ECLIA	26
Khan, Z. D [42].	Tanzania	East Africa	2023	cross-sectional	320	57.8	32.8	67.2	< 220 pmol/L	Serum vitamin B12	ECLIA	40
Mohammed, A. O. and A. A. Abd Elkarim [43]	Sudan	East Africa	2021	case‒control	50	54	48	52	NR	Serum vitamin B12	ELISA	9
Mahmoud, S. H. et al. [36]	Libya	North Africa	2025	Retrospective	281	50.79	39.6	60.4	<200 pg/mL	Serum vitamin B12	NR	67
Osama, H. et al. [26]	Egypt	North Africa	2016	cross-sectional	30	49.8	26.1	73.9	<180 pg/mL	Serum vitamin B12	ELISA	19
Fakkar, N. F. H. et al. [29]	Egypt	North Africa	2022	cross-sectional	50	53.2	36	64	<200 pg/mL	Serum vitamin B12	ECLIA	2
Elsayed, A. M. et al. [28]	Egypt	North Africa	2022	cross-sectional	100	60.21	49	51	<200 pg/mL	Serum vitamin B12	ECLIA	10
Elsaier, A. et al. [27]	Libya	North Africa	2017	cross-sectional	500	58.6	35	65	<200 pg/mL	Serum vitamin B12	NR	37
Yakubu, M. et al. [35]	Ghana	West Africa	2019	cross-sectional	196	50.4	30.1	69.9	<100 pg/mL + MMA ≥0.4 μmol/L	Serum vitamin B12 and MMA	ELISA	28
Naidoo, Pillay et al. [32]	South Africa	South Africa	2024	Retrospective	334	NR	25.2	74.8	<148 pmol/L	Serum vitamin B12 only	CLIA	6
Assoudani et al. [37]	Libya	North Africa	2026	Cross-sectional	37	NR	51.4	48.6	<200 pg/mL	Serum vitamin B12 only	CLIA	6
Sulaiman et al. [38]	Libya	North Africa	2023	Cross-sectional	150	56.24	34.7	65.3	<200 pg/mL	Serum vitamin B12 only	CLIA	31

Note: MMA: methylmalonic acid, CLIA: chemiluminescent immunoassay, ECLIA: electrochemiluminescence immunoassay, ELISA: enzyme-linked immunosorbent assay, HPLC: high-performance liquid chromatography, 4cB12: combined indicator of vitamin B12 status.

### 3.3 Pooled prevalence of vitamin B12 deficiency in Africa

Nineteen published studies comprising 3108 metformin-treated adults with T2DM were included in the meta-analysis of vitamin B12 deficiency. The reported prevalence of vitamin B12 deficiency ranged from 1.8% to 63.3%, with the lowest estimate reported in a study conducted in South Africa [32] and the highest in a study conducted in Egypt [28]. Substantial between-study heterogeneity was observed (I^2^ = 94.69%, τ^2^ = 0.99, p < 0.001). Using a random-effects model estimated by REML, the pooled prevalence of vitamin B12 deficiency among the available African studies was 14.7% (95% CI: 9.7–21.6%), with a corresponding 95% PI of 2.0–59.7% (Fig. 2). The wide PI indicates considerable variation in the expected prevalence across different clinical and population settings. Given the substantial between-study heterogeneity, this pooled estimate should be interpreted cautiously and as a summary of the available evidence rather than as a uniform estimate for the African population.

**Fig. 2:**
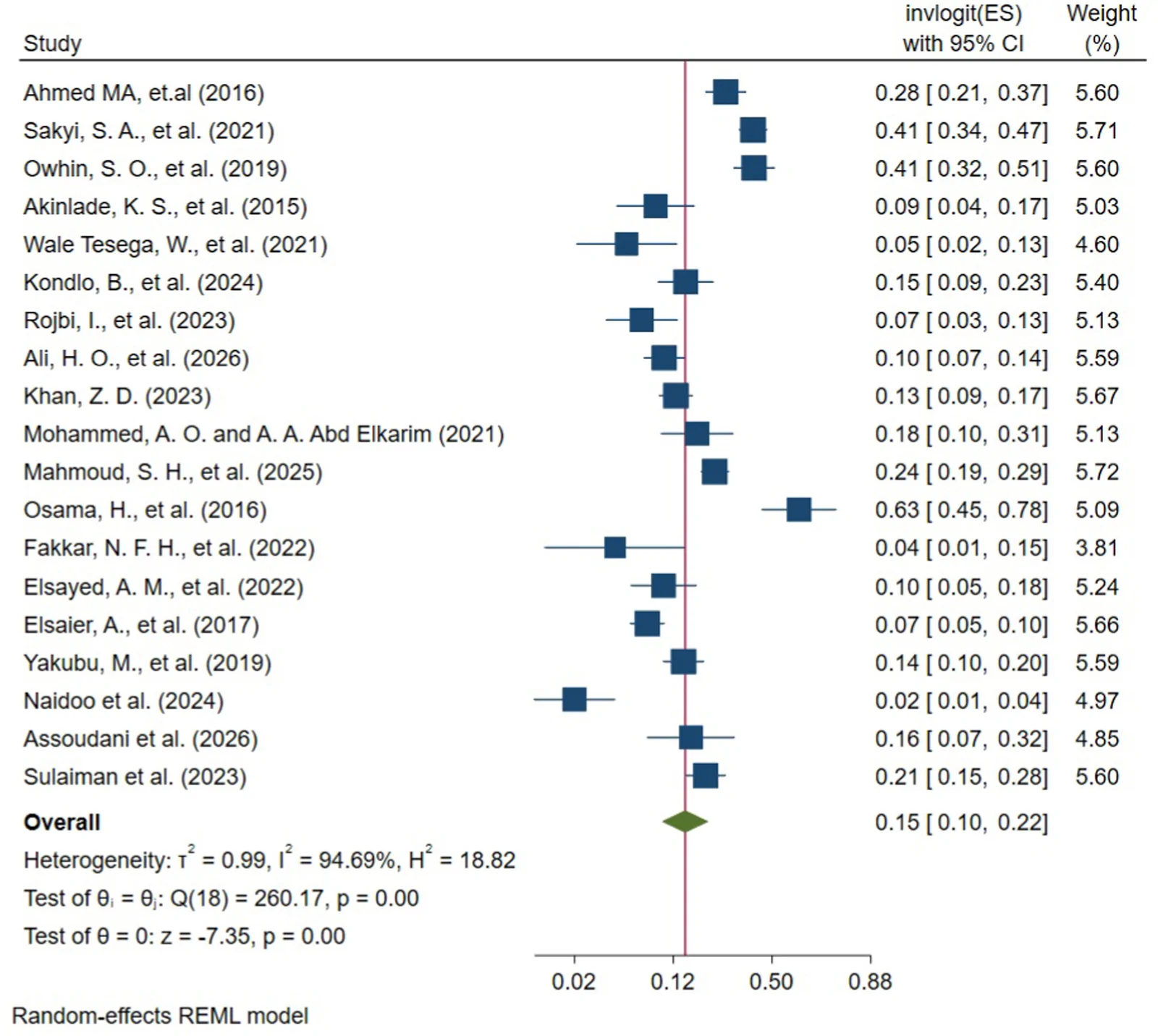
Forest plot of pooled vitamin B12 deficiency prevalence among metformin-treated adults with type 2 diabetes mellitus in African studies. Pooled prevalence: 14.7% (95% CI: 9.7–21.6%); 95% prediction interval: 2.0–59.7%.

### 3.4 Assessment of small-study effects

Visual inspection of the funnel plot suggested some asymmetry (Fig. 3). Potential small-study effects were assessed using Egger's regression test and Begg's rank correlation test. Egger's test indicated statistically significant evidence of small-study effects (z = −2.11, p = 0.0345), whereas Begg's test did not show statistically significant evidence (Kendall's score = −37.00, z = −1.33, p = 0.2079). These findings were interpreted cautiously because, although 19 studies were included, between-study heterogeneity remained substantial. In meta-analyses of proportions, funnel-plot asymmetry may also arise from differences in study size, prevalence, methodological characteristics, and the transformation of proportions, rather than small-study effects alone. Therefore, the discordant results of Egger's and Begg's tests should be considered exploratory evidence of potential small-study effects rather than definitive evidence for the presence or absence of small-study effects.

**Fig. 3:**
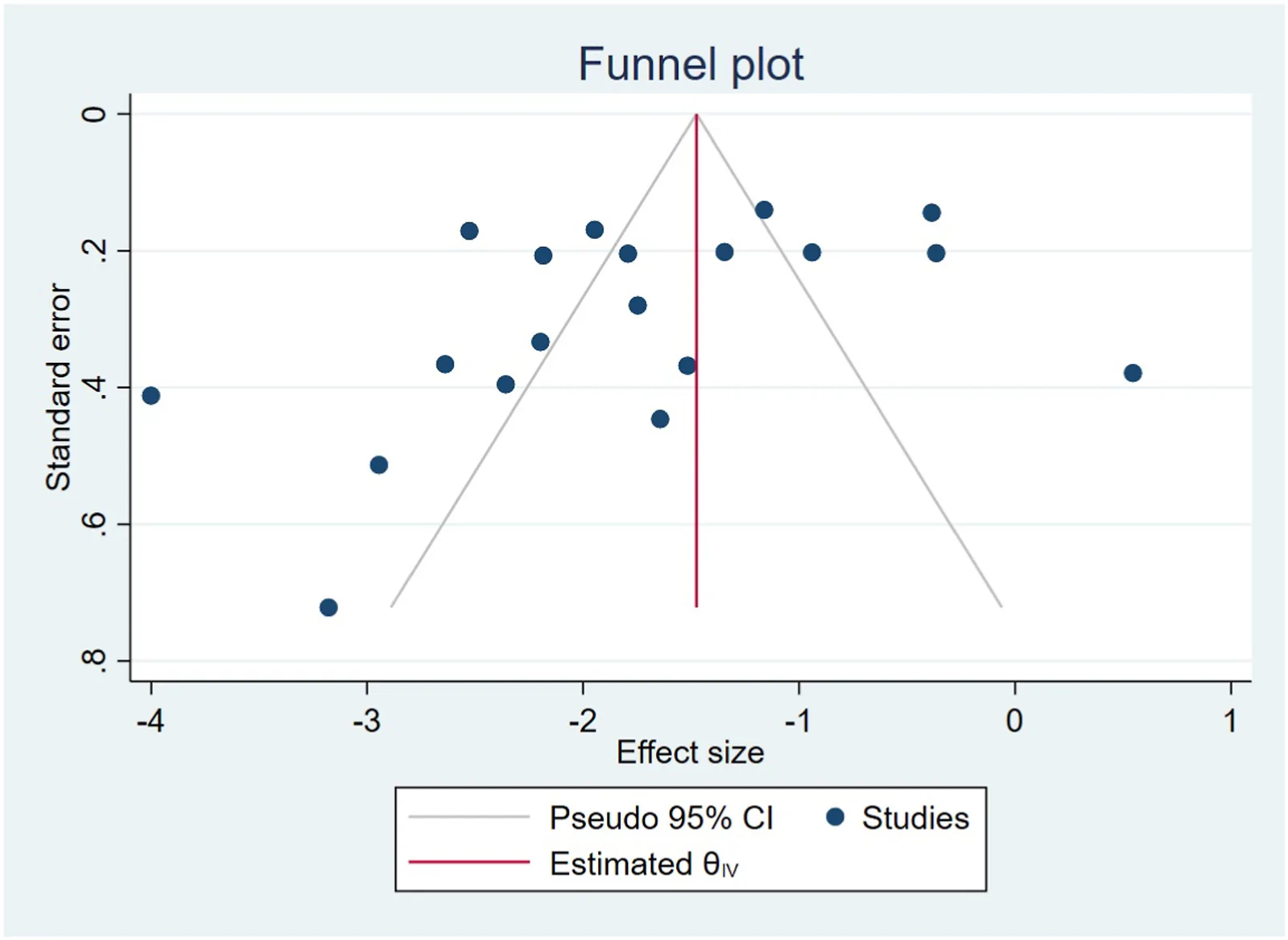
Funnel plot assessing potential small-study effects in the meta-analysis of vitamin B12 deficiency among metformin-treated adults with T2DM. Note. T2DM: Type 2 diabetes mellitus.

### 3.5 Sensitivity analysis

Sensitivity analyses were conducted to assess the robustness of the pooled prevalence estimate to individual studies, methodological concerns, study design, and diagnostic thresholds. First, a leave-one-out sensitivity analysis was performed by sequentially excluding each study from the meta-analysis. The resulting pooled prevalence estimates ranged from 13% to 16%, compared with the overall pooled prevalence of 14.7%, indicating that no single study had a substantial influence on the pooled estimate or materially altered its overall interpretation. These findings suggest that the pooled estimate was relatively stable with respect to the contribution of individual studies (Fig. 4).

**Fig. 4:**
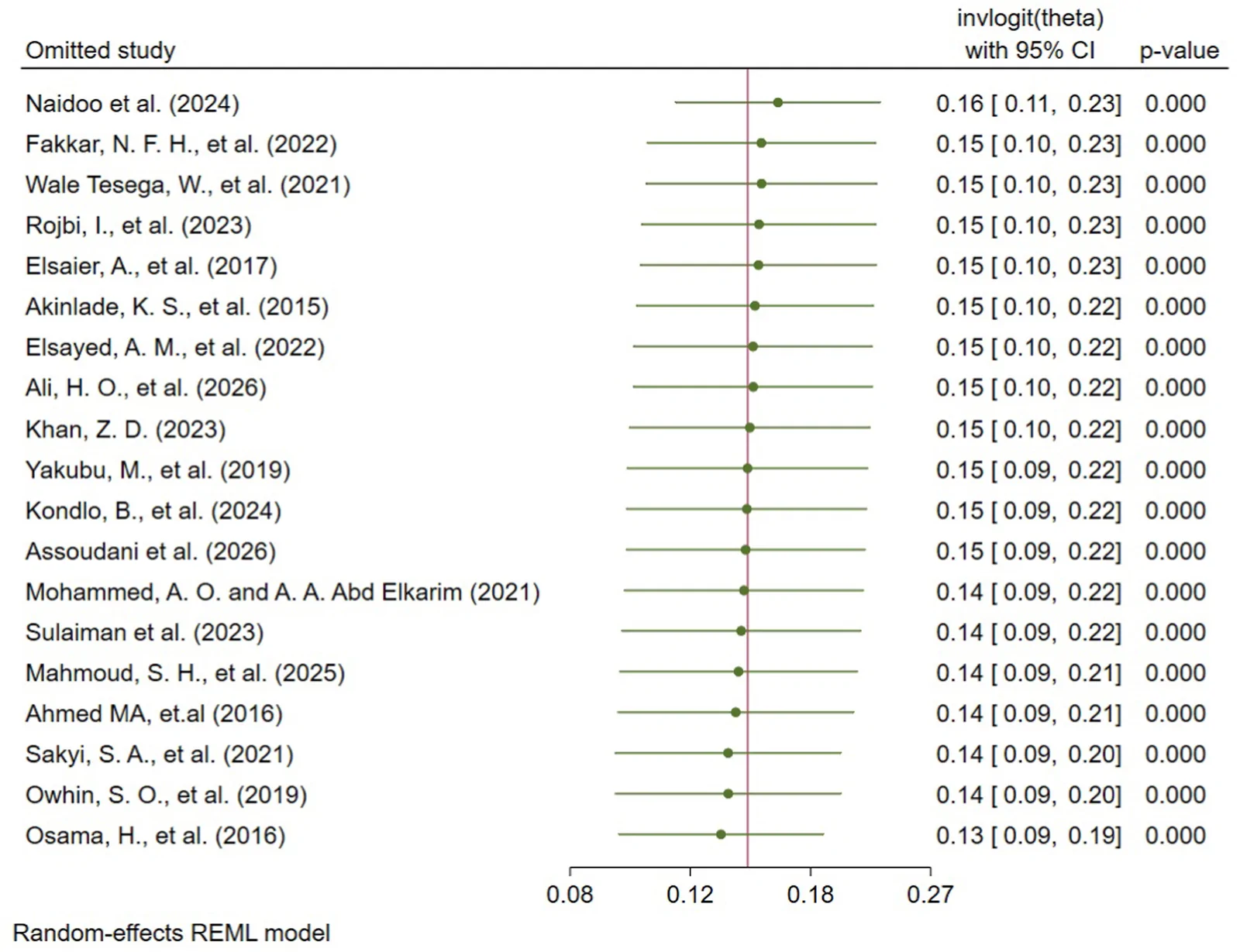
Leave-one-out sensitivity analysis of the pooled prevalence of vitamin B12 deficiency among metformin-treated adults with T2DM. Note. T2DM: Type 2 diabetes mellitus.

Second, sensitivity analyses were performed to examine the potential influence of the two studies described by their authors as case-control studies. After excluding these two studies, the pooled prevalence was 13.4% (95% CI: 8.6–20.3%) across 17 studies involving 2958 participants. When the analysis was further restricted to the 14 cross-sectional studies, the pooled prevalence was 14.5% (95% CI: 9.2–22.1%) among 2242 participants. Substantial between-study heterogeneity remained in both analyses (I^2^ = 94.72% and 93.69%, respectively). Thus, although exclusion of the case-control studies resulted in a modest reduction in the pooled prevalence estimate, substantial heterogeneity persisted, indicating that study design alone did not explain the observed variability between study estimates.

Finally, to assess the influence of heterogeneity in diagnostic definitions, a threshold-restricted sensitivity analysis was conducted among 11 studies using approximately comparable serum vitamin B12 deficiency thresholds (197.00–203.31 pg/mL). The pooled prevalence was 11.7% (95% CI: 6.5–20.2%), with considerable between-study heterogeneity (I^2^ = 93.1%; τ^2^ = 1.0661; Cochran's Q = 145.09, p < 0.001). This estimate was modestly lower than that obtained in the primary analysis of all 19 studies (14.7%; 95% CI: 9.7–21.6%). However, substantial heterogeneity persisted despite restricting the analysis to studies using approximately comparable diagnostic thresholds, suggesting that differences in serum vitamin B12 cutoffs alone did not fully explain the observed between-study variability.

Overall, the sensitivity analyses indicate that the pooled prevalence estimate remained broadly similar after excluding individual studies, alternative study-design specifications, and studies using different diagnostic thresholds. However, substantial heterogeneity persisted across these analyses, underscoring the need for cautious interpretation of the pooled estimate.

### 3.6 Assessment of heterogeneity

A Galbraith (radial) plot was used to further explore between-study heterogeneity (Fig. 5). Several studies lay outside the 95% confidence limits, indicating that multiple studies contributed significantly to the observed heterogeneity. This wide dispersion is consistent with the very high statistical heterogeneity observed in the meta-analysis (I^2^ = 94.69%) and complements the sensitivity analysis, which showed that sequential exclusion of individual studies did not materially alter the pooled prevalence estimate.

**Fig. 5:**
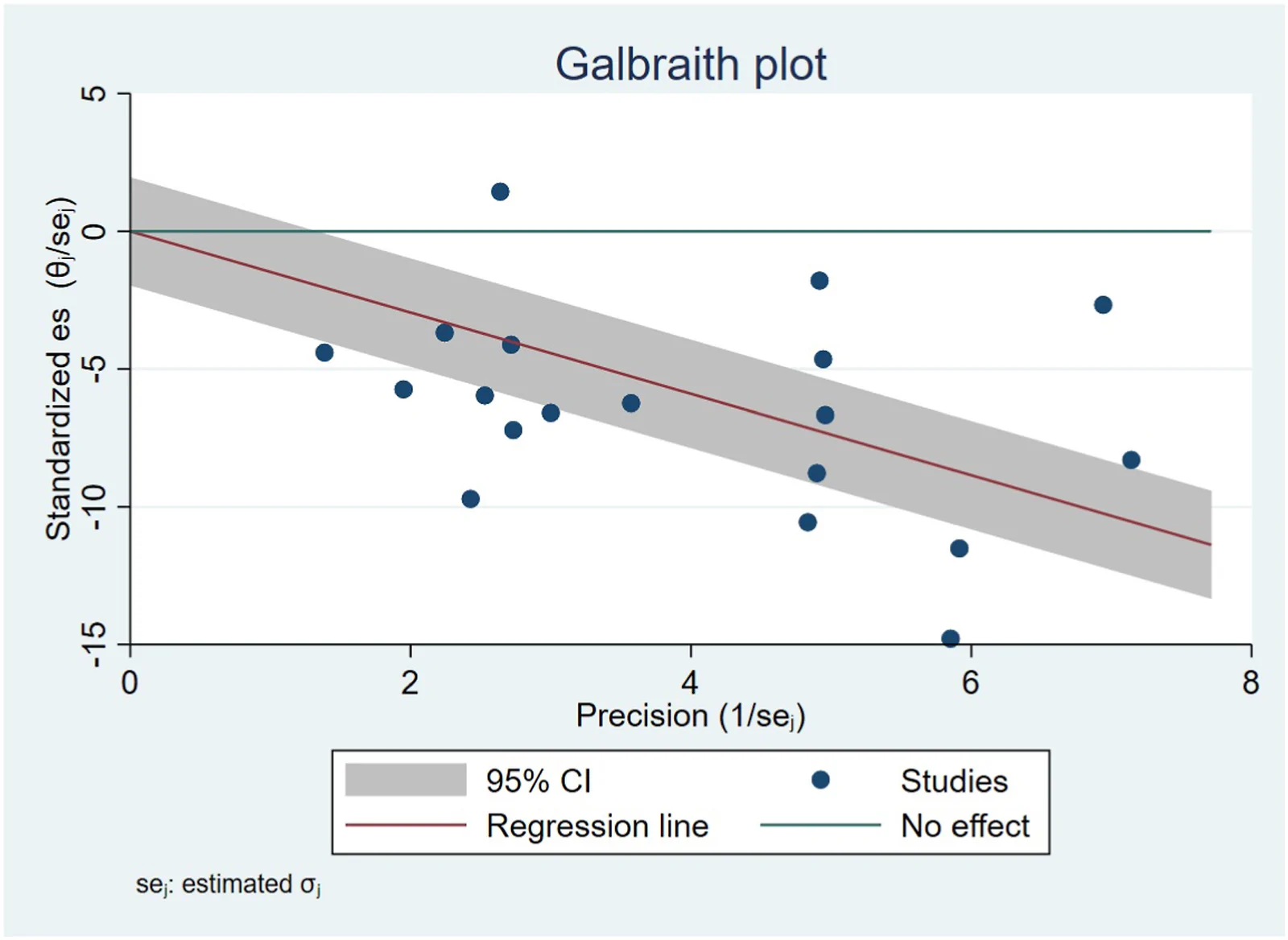
Galbraith (radial) plot assessing between-study heterogeneity.

### 3.7 Subgroup analysis

Subgroup analyses were performed using predefined characteristics, including African region, laboratory assay, diagnostic cutoff value, and publication year, to explore potential sources of heterogeneity. Pooled prevalence estimates with 95% CIs and heterogeneity statistics (I^2^) are presented for subgroups with sufficient numbers of contributing studies, while single-study categories are presented descriptively in Table 2. Several subgroup estimates were based on a small number of studies and should therefore be interpreted cautiously.

**Table 2:** Subgroup analysis of vitamin B12 deficiency prevalence among metformin-treated adults with type 2 diabetes mellitus.

Subgroup	No. of studies	Sample size	No. of cases	Pooled prevalence (95% CI)	I^2^ (%)
By region					
East Africa	4	707	79	11.5% (8.9–14.7%)	49.4
North Africa	8	1268	180	15.1% (7.6–27.9%)	92.1
South Africa	3	556	55	10.0% (1.8–39.5%)	95.5
West Africa	4	577	157	23.3% (10.4–44.5%)	94.2
By publication year					
Before 2020	6	1028	166	22.3% (10.0–42.5%)	95.5
2020 and after	13	2080	305	12.0% (7.6–18.4%)	92.0
By diagnostic assay					
CLIA	5	722	81	10.6% (3.7–26.5%)	92.4
ECLIA	5	828	93	11.5% (9.5–13.9%)	13.2
ECLIA + ELISA	1	200	81	Not pooled; study-specific prevalence = 40.5%	N.A.
ELISA	4	376	97	31.1% (13.7–56.2%)	93.1
HPLC	1	81	7	Not pooled; study-specific prevalence = 8.6%	N.A.
By diagnostic cutoff					
Serum vitamin B12 threshold <148 pmol/L	11	1801	238	13.4% (7.9–21.6%)	93.2
Serum vitamin B12 threshold ≥148 pmol/L	3	555	81	14.9% (1.9–60.9%)	98.3
Alternative/non-comparable definitions	5	752	152	17.4%(9.7–29.4%)	90.8

Note: CLIA: chemiluminescent immunoassay; ECLIA: electrochemiluminescence immunoassay; ELISA: enzyme-linked immunosorbent assay; HPLC: high-performance liquid chromatography; N.A., not available; subgroup analyses were based on studies with available information for the respective characteristic. Subgroup estimates based on fewer than three studies should be interpreted cautiously and are presented descriptively. These estimates should not be interpreted as evidence of between-group differences.

Pooled prevalence estimates varied across regions, diagnostic assays, publication years, and diagnostic cutoff definitions. Regional estimates varied across subgroups; however, these analyses were exploratory and do not establish differences in prevalence between regions. Similarly, estimates according to laboratory assay should not be interpreted as evidence of assay-related differences because some assay strata contained only one or a small number of studies. The pooled prevalence was 23.3% (95% CI: 10.4–44.5%) in West Africa and 10.0% (95% CI: 1.8–39.5%) in South Africa. Studies published before 2020 had a pooled prevalence of 22.3% (95% CI: 10.0–42.5%), compared with 12.0% (95% CI: 7.6–18.4%) among studies published from 2020 onward. The pooled prevalence was 11.5% (95% CI: 9.5–13.9%) among studies using ECLIA and 31.1% (95% CI: 13.7–56.2%) among those using ELISA. The ECLIA + ELISA and HPLC categories each comprised a single study and were therefore not pooled; their study-specific prevalence estimates were 40.5% and 8.6%, respectively. These single-study estimates are presented descriptively and should not be interpreted as evidence of differences associated with assay type.

For the diagnostic cutoff analysis, reported vitamin B12 thresholds were retained as specified by the original studies, with thresholds reported in pg/mL converted to pmol/L using 1 pg/mL = 0.738 pmol/L, and 1 pg/dL = 0.00738 pmol/L for subgroup classification. Of the 19 studies, 11 used serum vitamin B12 thresholds <148 pmol/L, 3 used thresholds ≥148 pmol/L, and 5 used alternative or non-comparable definitions, including 4cB12, combined serum vitamin B12 and MMA, an unreported threshold, or a non-comparable unit definition. The pooled prevalence was 13.4% (95% CI: 7.9–21.6%; I^2^ = 93.2%) among the 11 studies using serum vitamin B12 thresholds <148 pmol/L and 14.9% (95% CI: 1.9–60.9%; I^2^ = 98.3%) among the 3 studies using thresholds ≥148 pmol/L. The five studies using alternative or non-comparable definitions yielded a pooled prevalence of 17.4% (95% CI: 9.7–29.4%; I^2^ = 90.8%). The test for subgroup differences was not statistically significant (Qb = 0.48, df = 2, P = 0.788).

Most subgroup analyses showed substantial heterogeneity (I^2^ >90%), except for the East Africa and ECLIA subgroups (I^2^ = 49.4% and 13.2%, respectively) (Table 2). Overall, these subgroup findings should be considered exploratory given the small number of studies in several strata and the substantial residual heterogeneity. The observed variation across subgroups should not be interpreted as evidence of genuine regional, assay-related, publication-period, or cutoff-related differences in vitamin B12 deficiency prevalence without adequate study numbers and formal statistical evidence of subgroup interaction.

### 3.8 Meta-regression

Univariate meta-regression analyses were performed to explore whether selected study-level characteristics were associated with variation in vitamin B12 deficiency prevalence. None of the examined moderators was statistically significant: laboratory assay (p = 0.124), publication year (β = −0.095, p = 0.205), sample size (β = −0.0026, p = 0.195), mean/median age (β = −0.055, p = 0.280), and geographic region (Wald χ^2^ = 2.06, p = 0.560). Laboratory assay showed the largest estimated reduction in between-study variance (pseudo-R^2^ = 17.37%), although this was not statistically significant. Given the small number of studies (n = 19), these findings are exploratory, and non-significant associations should not be interpreted as evidence that the examined characteristics do not contribute to the observed heterogeneity. Pseudo-R^2^ values are reported descriptively and were not interpreted as evidence of explanatory importance (Table 3).

**Table 3:** Exploratory univariate meta-regression analyses of study-level moderators.

Covariates	Β (95% ci)/wald χ^2^	Standard error	P value	Pseudo-r^2^ (%)
Publication year	−0.095 (−0.246 to 0.057)	0.072	0.205	3.90
Sample size	−0.0026 (−0.0067 to 0.0015)	0.0019	0.195	6.50
Mean/Median age	−0.055 (−0.159 to 0.050)	0.049	0.280	2.61
Region	Wald χ^2^ = 2.06	---	0.560	−6.52
Laboratory assay	Wald χ^2^ = 8.65	---	0.124	17.37

**Note:** Meta-regression analyses were exploratory and based on only 19 studies. Pseudo-R^2^ values are presented descriptively and should not be interpreted as evidence of explanatory importance. Nonsignificant moderator associations do not establish that the corresponding study-level characteristics are unrelated to the observed heterogeneity. Detailed coding schemes, reference categories, study counts, and regression coefficients for categorical moderators are provided in Supplementary Table (SF6).

### 3.9 Factors associated with vitamin B12 deficiency

Several factors were reported to be associated with vitamin B12 deficiency among metformin-treated adults with T2DM. Because relatively few studies reported adjusted effect estimates and substantial heterogeneity existed in exposure definitions, reference categories, and covariate-adjustment strategies, quantitative synthesis of these associations was not considered appropriate. The individual study findings are summarized in Supplementary Table (SF7).

Longer duration of metformin therapy and higher metformin dose were among the factors most frequently associated with vitamin B12 deficiency across the included studies. However, these findings should not be interpreted as evidence of a definitive dose–response relationship, as most available evidence was derived from observational studies with cross-sectional or retrospective designs and differing exposure categorizations. In addition, cumulative metformin exposure may be correlated with duration of diabetes and other clinical characteristics, while renal function, dietary vitamin B12 intake, vitamin supplementation, and concomitant use of acid-suppressive medications may also influence vitamin B12 status and potentially confound these associations. Older age, poor glycemic control, and higher body fat were also reported in individual studies, but these associations were not consistently evaluated across studies. Therefore, the available evidence supports longer metformin exposure and higher metformin dose as potentially associated factors rather than demonstrating an independent causal or dose–response relationship.

## 4 Discussion

This systematic review and meta-analysis estimated a pooled prevalence of vitamin B12 deficiency of 14.7% (95% CI: 9.7–21.6%) among metformin-treated adults with T2DM in Africa. This pooled estimate falls within the range reported by several individual studies from African and non-African settings, where prevalence has generally ranged from approximately 9% to 18% [31], [35], [42], [43], [44], [45], [46], [47], [48], although higher prevalence estimates have also been reported in some African and Asian populations [26], [30], [33], [34], [36], [49], [50]. Previous global meta-analyses have reported higher pooled prevalence estimates of 23.2% and 28.7% among metformin-treated patients [20], [51], while a more recent systematic review and meta-analysis published in September 2026 reported a pooled prevalence of 32.1% [52]. Differences between the present findings and these estimates may reflect variations in geographic coverage, study populations, diagnostic criteria, laboratory methods, and metformin exposure, limiting direct comparability across reviews.

Importantly, the included studies used different approaches to defining vitamin B12 deficiency, including conventional serum vitamin B12 measurements and functional biomarkers such as MMA and 4cB12, which are not interchangeable measures of vitamin B12 status. The influence of diagnostic definitions is further illustrated by a recent Danish cross-sectional study published in August 2026, which reported a vitamin B12 deficiency prevalence of 2.3% among metformin-treated adults with T2DM using a combined biochemical definition incorporating plasma vitamin B12 and MMA [53]. Differences in cutoff values, laboratory assays, dietary vitamin B12 intake, nutritional status, age, comorbidities, and healthcare practices may therefore contribute to the observed between-study variation. Consequently, differences in reported prevalence across African settings may partly reflect diagnostic and methodological variation rather than true epidemiological differences. The pooled estimate should therefore be interpreted as a synthesis of study-defined vitamin B12 deficiency rather than as an estimate based on a single standardized diagnostic approach.

To further assess the potential influence of diagnostic thresholds, we conducted a sensitivity analysis restricted to 11 studies using serum vitamin B12 thresholds approximately corresponding to 200 pg/mL. The pooled prevalence was 11.7% (95% CI: 6.5–20.2%), compared with 14.7% (95% CI: 9.7–21.6%) in the primary analysis. Despite this modest reduction, substantial heterogeneity persisted (I^2^ = 93.1% versus 94.69% in the primary analysis). These findings suggest that differences in serum vitamin B12 thresholds alone are unlikely to account for the substantial between-study heterogeneity, which may also reflect differences in laboratory methods, study populations, nutritional factors, diabetes characteristics, and metformin exposure.

Despite being lower than estimates from some global analyses, the pooled prevalence of 14.7% remains clinically relevant, indicating that vitamin B12 deficiency is not uncommon among metformin-treated adults with T2DM in the available African evidence. However, substantial between-study heterogeneity and limited geographical coverage mean that this estimate should not be interpreted as a uniform prevalence across African populations.

Substantial between-study heterogeneity was observed (I^2^ = 94.69%), and the 95% PI ranged from 2.0% to 59.7%. This wide PI indicates that the prevalence in a new, comparable setting could differ substantially from the pooled estimate. The observed heterogeneity is likely to reflect differences in diagnostic criteria, laboratory methods, patient characteristics, metformin exposure, and clinical and healthcare contexts. Therefore, the pooled prevalence should be interpreted as a summary estimate of the available evidence rather than as a continent-wide prevalence.

Subgroup analyses showed variation in vitamin B12 deficiency prevalence across geographic settings, publication periods, laboratory assays, and diagnostic criteria. However, these findings should be interpreted cautiously because several subgroups contained few studies and substantial heterogeneity persisted within many strata. In particular, the ECLIA-plus-ELISA subgroup was based on a single study and therefore does not provide sufficient evidence for an assay-specific difference. Similarly, differences observed across geographic regions, publication periods, or diagnostic categories should be regarded as descriptive patterns rather than evidence of genuine moderator effects. None of the examined moderators showed a statistically significant association with prevalence in the univariate meta-regression analyses.

Univariate meta-regression found no statistically significant associations between prevalence and publication year, sample size, age, geographic region, or laboratory assay. These findings should be interpreted cautiously because only 19 studies were available, limiting the statistical power to detect moderator effects and increasing the potential for unstable estimates. Therefore, the absence of statistically significant associations does not demonstrate that the examined study-level characteristics are unrelated to the substantial between-study heterogeneity. In addition, because the moderators were assessed at the study level rather than the individual-participant level, the findings may be subject to ecological bias and should not be interpreted as individual-level associations. Pseudo-R^2^ estimates were not interpreted as evidence of explanatory importance because of the small number of studies and the exploratory nature of the meta-regression analyses.

Potential sources of residual heterogeneity include differences in dietary vitamin B12 intake, nutritional status, socioeconomic status, adherence to metformin therapy, metformin dose and duration, concomitant use of medications that may impair vitamin B12 absorption, such as proton pump inhibitors and H2-receptor antagonists, vitamin supplementation, diabetes severity, healthcare access, and routine screening practices. Nutritional status and exposure to nutraceuticals or vitamin supplements may also influence micronutrient-related outcomes and should be considered when interpreting treatment-associated effects. Evidence on nutraceutical–drug interactions suggests that concomitant use of nutraceuticals can modify treatment effects in some pharmacological contexts, although the available evidence is not specific to metformin or vitamin B12 [54]. These factors were insufficiently reported or inconsistently measured in the included studies and therefore could not be quantitatively evaluated in the present meta-analysis. Methodological differences that were not consistently reported across studies may also have contributed to the observed heterogeneity.

The pooled estimate was not materially altered by the exclusion of any single study in the leave-one-out sensitivity analysis, supporting the stability of the overall estimate. However, this stability should be interpreted alongside the substantial heterogeneity and wide PI. The stability of the pooled estimate indicates that the overall summary was not driven by a single study but does not imply that prevalence is similar across settings.

The inclusion of two studies described by their authors as case-control studies is another consideration when interpreting the pooled prevalence estimate. Although these studies did not select participants according to vitamin B12 deficiency status and calculated prevalence within the metformin-treated groups, their hospital-based and exposure-defined sampling differs from conventional cross-sectional prevalence sampling and may limit the generalizability of their estimates. In Owhin et al., the prevalence estimate was based on 100 metformin-treated participants within an overall study population of 200, while Mohammed and Abd Elkarim reported the prevalence among 50 metformin-treated participants within an overall study population of 100. Sensitivity analyses excluding these two studies yielded a pooled prevalence of 13.4% (95% CI: 8.6–20.3%) across 17 studies, while restriction to the 14 cross-sectional studies yielded a pooled prevalence of 14.5% (95% CI: 9.2–22.1%). Substantial heterogeneity persisted in both analyses (I^2^ = 94.72% and 93.69%, respectively). Thus, exclusion of the two case-control studies resulted in a modest reduction in the pooled prevalence estimate but did not substantially reduce the observed between-study heterogeneity. These findings indicate that the inclusion of these two studies was not sufficient to account for the high heterogeneity observed in the primary analysis.

An important consideration is the geographical distribution of the available evidence. Although this review focused on Africa, the included studies were derived from a limited number of African countries and were not evenly distributed across the continent. Consequently, the pooled prevalence may not adequately represent countries or regions for which evidence is currently unavailable. Differences in dietary patterns, nutritional status, healthcare systems, access to laboratory testing, metformin prescribing practices, and screening practices across African settings may further limit the generalizability of the pooled estimate.

Several studies investigated factors associated with vitamin B12 deficiency among metformin-treated adults with T2DM, but the available evidence was insufficient to permit quantitative synthesis. Only a small number of studies reported adjusted effect estimates, and substantial variability existed in exposure definitions, reference categories, and covariate adjustment. Consequently, pooling these estimates would not have been methodologically appropriate. Nevertheless, the available evidence suggests that longer duration of metformin therapy, higher metformin dose, older age, and poor glycemic control may be associated with vitamin B12 deficiency. More broadly, supplement and nutraceutical exposure may influence pharmacological outcomes [54], although this evidence does not specifically establish an association with vitamin B12 status or metformin-associated deficiency. However, these potential influences were not consistently evaluated in the included studies. These findings should be interpreted cautiously because they are based on a limited number of heterogeneous observational studies and do not establish independent causal associations.

Overall, the findings indicate that vitamin B12 deficiency represents a potentially important clinical concern among metformin-treated adults with T2DM in Africa, although its prevalence varies substantially across settings. The high heterogeneity, wide PI, and geographical gaps in the available evidence underscore the need for standardized diagnostic approaches and better-designed multicenter studies. Future research should establish consistent vitamin B12 deficiency thresholds, harmonize laboratory methods, and systematically assess metformin dose and duration, dietary intake, concomitant medications, supplementation, and other relevant clinical and socioeconomic factors. Such evidence would improve estimation of the burden of vitamin B12 deficiency and help inform context-specific screening and management strategies for people with T2DM receiving metformin therapy.

### 4.1 Limitations of the study

This review has several limitations. Substantial between-study heterogeneity was observed, likely reflecting differences in study populations, diagnostic thresholds, laboratory assays, and study designs. Two included studies used hospital-based, exposure-defined case-control sampling, which may limit the generalizability of their prevalence estimates; however, excluding these studies produced only modestly lower pooled estimates, with substantial heterogeneity persisting. Diagnostic-threshold heterogeneity and inconsistent reporting of vitamin B12 units may have further contributed to variation in prevalence estimates.

The observational nature of the included studies limits causal inference, while the English-language restriction, limited number of studies, and uneven geographical representation may reduce generalizability and introduce selection or language bias. Measurement of metformin dose and duration, supplementation, dietary intake, renal function, and acid-suppressive medication use was inconsistent or insufficient, limiting assessment of their potential influence on vitamin B12 status. Quantitative synthesis of associated factors was therefore not feasible because of differences in exposure definitions, reference groups, and covariate adjustment. Potential small-study effects cannot be excluded, and meta-regression findings should be interpreted cautiously given the small number of studies and the ecological nature of study-level analyses.

## 5 Conclusions and recommendations

Vitamin B12 deficiency was frequently reported among metformin-treated adults with T2DM in the included African studies, which yielded a pooled prevalence of 14.7% (95% CI: 9.7–21.6%). However, substantial heterogeneity and variation in diagnostic definitions limit interpretation as a uniform or continent-wide prevalence. A sensitivity analysis restricted to 11 studies using approximately comparable serum vitamin B12 thresholds yielded a similar prevalence of 11.7% (95% CI: 6.5–20.2%), while heterogeneity remained very high, suggesting that threshold variation alone does not explain the observed variability.

Evidence on associated factors was insufficient for quantitative synthesis, although longer metformin duration, higher doses, older age, and poor glycemic control were reported as potential factors. Vitamin B12 assessment may be particularly relevant for patients receiving long-term or high-dose metformin, especially those with neurological or hematological manifestations or other risk factors for deficiency. However, this meta-analysis did not evaluate effectiveness, cost-effectiveness, or the optimal screening interval; therefore, the findings support consideration of risk-based or targeted assessment rather than universal screening. Future multicenter and prospective studies should use standardized diagnostic methods, systematically assess metformin exposure and other risk factors, and evaluate the effectiveness and cost-effectiveness of different monitoring strategies and screening intervals.

## CRediT authorship contribution statement

**Kibrom Aregawi Amare:** Writing – review & editing, Writing – original draft, Visualization, Validation, Supervision, Software, Resources, Project administration, Methodology, Investigation, Formal analysis, Data curation, Conceptualization. **Niguse Shishay Kahsay:** Writing – review & editing, Methodology, Investigation, Conceptualization. **Bahlbi Hailay Abraha:** Writing – review & editing, Investigation, Data curation, Conceptualization. **Yemane Mengsteab Hagos:** Writing – review & editing, Software, Investigation, Formal analysis, Conceptualization. **Merhawi Alemu Birhanu:** Writing – review & editing, Methodology, Data curation, Conceptualization. **Abel Deres Teka:** Writing – review & editing, Methodology, Formal analysis, Conceptualization. **Mebrahtu Tefera Aynalem:** Writing – review & editing, Methodology, Formal analysis, Conceptualization.

## Ethics declaration

Ethical approval and informed consent were not required because this study was a systematic review and meta-analysis of data extracted from previously published studies. The authors did not recruit or directly interact with human participants, collect new participant data or biological materials, or access identifiable individual-level information. Ethical approval and informed consent were therefore not required for this secondary analysis of published data.

This study was performed in compliance with relevant laws, regulatory frameworks and guidelines where the research took place. Ethics committee approval was not required under relevant laws and institutional guidelines. Ethical approval was not required because this study was a systematic review and meta-analysis of data from previously published studies and did not involve direct recruitment or participation of human subjects, collection of biological materials, or access to identifiable personal data.

## Ethical approval and consent

Not applicable. This study synthesized data from previously published studies and did not involve direct participation of human subjects.

## Consent for publication

Not applicable.

## Data availability

The data extracted from the included studies and used in the meta-analysis are provided in Supplementary Additional File S4. Additional information is available from the corresponding author upon reasonable request.

## Declaration of generative AI in scientific writing

During the preparation of this work, the authors used AI tool to assist with language refinement, grammar, and clarity of presentation. All AI-assisted content was critically reviewed and edited by the authors, who take full responsibility for the accuracy, integrity, and final content of the published work.

## Funding

The authors received no specific funding for this study.

## Declaration of competing interests

The authors declare that they have no competing interests.

## Abbreviations

T2DM: type 2 diabetes mellitus; CI: confidence interval; I^2^: I-squared statistic; AJOL: African Journals Online; AIM: African Index Medicus; DOAJ: Directory of Open Access Journals; CLIA: chemiluminescent immunoassay; ECLIA: electrochemiluminescence immunoassay; ELISA: enzyme-linked immunosorbent assay; HPLC: high-performance liquid chromatography; PRISMA: Preferred Reporting Items for Systematic Reviews and Meta-Analyses.

## Appendix A Supplementary data

S1 PRISMA 2020 checklist for reporting systematic reviews and meta-analyses.

S2 Database-specific search strategies used in the systematic review.

S3 Quality assessment of the included studies.

S4 Data extracted from the included studies and used for the meta-analysis.

S5 Study-specific diagnostic thresholds for vitamin B12 deficiency, standardized values, and classification for threshold-restricted sensitivity analysis.

S6 Coding schemes, study counts, and meta-regression coefficients for categorical moderators.

S7 Factors associated with vitamin B12 deficiency among metformin-treated adults with type 2 diabetes mellitus.
