# Supplementary material for: Vitamin B12 deficiency in metformin-treated adults with type 2 diabetes mellitus in Africa: A systematic review and meta-analysis
Source: Metabol Open. 2026 Nov 2;32:100524. doi: 10.1016/j.metop.2026.100524 (PMC13760081; doi:10.1016/j.metop.2026.100524)
Supplement: Supplementary file 2 [file mmc2.docx]

**Supplementary File (SF2). Database-specific search strategies**

**Summary of database searches**

| **Database/source** | **Platform** | **Date searched** | **Search strategy** | **Filters/limits** | **Records retrieved** |
| --- | --- | --- | --- | --- | --- |
| PubMed/MEDLINE | PubMed | 18 October 2026 | Full strategy below | None | 282 |
| Cochrane Library | Cochrane Library | 18 October 2026 | Full strategy below | None | 67 |
| Google Scholar | Google Scholar | 18 October 2026 | Full strategy below | None | 1,550 |
| African Journals Online (AJOL) | AJOL | 18 October 2026 | Full strategy below | None | 1,811 |
| ScienceDirect | ScienceDirect | 18 October 2026 | Full strategy below | None | 1353 |
| Directory of Open Access Journals (DOAJ) | DOAJ | 18 October 2026 | Full strategy below | None | 154 |
| African Index Medicus (AIM) | AIM | 18 October 2026 | Full strategy below | None | 94 |

1. **PubMed/MEDLINE**

Date searched: 18 October 2026

Platform: PubMed

Limits: None

Records retrieved: n = 282

Full search string

("Vitamin B12 Deficiency"[Mesh] OR "Vitamin B 12"[Mesh] OR "vitamin B12"[tiab] OR "vitamin B-12"[tiab] OR "vitamin B 12"[tiab] OR cobalamin[tiab] OR cyanocobalamin[tiab] OR "B12 deficiency"[tiab]) AND ("Metformin"[Mesh] OR metformin[tiab] OR glucophage[tiab]) AND ("Diabetes Mellitus, Type 2"[Mesh] OR "type 2 diabetes"[tiab] OR "type 2 diabetes mellitus"[tiab] OR "type II diabetes"[tiab] OR T2DM[tiab] OR "type 2 diabetic"[tiab] )

2. **Cochrane Library**

Date searched: 18 October 2026

Platform: Cochrane Library

Limits: None

Records retrieved: n = 67

Full search string

Step 1: Vitamin B12 deficiency terms

Search 1 MeSH descriptor Vitamin B12 Deficiency explode all trees

Search 2 MeSH descriptor Vitamin B 12 explode all trees

Search 3 vitamin NEXT B12:ti,ab,kw

Search 4 vitamin NEXT B-12:ti,ab,kw

Search 5 cobalamin:ti,ab,kw

Search 6 cyanocobalamin:ti,ab,kw

Search 7 (combine Vitamin B12 terms) #1 OR #2 OR #3 OR #4 OR #5 OR #6

Step 2: Metformin terms

Search 8 MeSH descriptor Metformin explode all trees

Search 9 metformin:ti,ab,kw

Search 10 glucophage:ti,ab,kw

Search 11 (combine Metformin terms) #8 OR #9 OR #10

Step 3: Type 2 Diabetes terms

Search 12 MeSH descriptor Diabetes Mellitus Type 2 explode all trees

Search 13 type NEXT 2 NEXT diabetes:ti,ab,kw

Search 14 type NEXT II NEXT diabetes:ti,ab,kw

Search 15 T2DM:ti,ab,kw

Search 16 (combine diabetes terms) #12 OR #13 OR #14 OR #15

Step 4: Final combination

Search 17 #7 AND #11 AND #16

3. **Google scholar**

Date searched: 18 October 2026

Platform: Google scholar

Limits: None

Records retrieved: n = 1550

Full search string

("vitamin B12 deficiency" OR "vitamin B-12 deficiency" OR cobalamin OR cyanocobalamin) AND (metformin OR glucophage) AND ("type 2 diabetes" OR "type II diabetes" OR T2DM) AND ("Africa" OR "African")

4. **African Journals Online (AJOL)**

Date searched: 18 October 2026

Platform: AJOL

Limits: None

Records retrieved: n = 1811

Full search string

metformin "vitamin B12 deficiency" "type 2 diabetes"

5. **ScienceDirect**

Date searched: 18 October 2026

Platform: ScienceDirect

Limits: None

Records retrieved: n = 1353

Full search string

("vitamin B12 deficiency" OR "vitamin B12" OR cobalamin) AND metformin AND ("type 2 diabetes" OR "type II diabetes" OR T2DM)

6. **Directory of Open Access Journals (DOAJ)**

Date searched: 18 October 2026

Platform: DOAJ

Limits: None

Records retrieved: n = 154

Full search string

metformin AND "vitamin B12"

7. **African Index Medicus (AIM)**

Date searched: 18 October 2026

Platform: **AIM**

Limits: None

Records retrieved: n = 94

Full search string

(vitamin B12 OR cobalamin OR "vitamin B12 deficiency") AND (metformin) AND ("type 2 diabetes" OR T2DM)
