# Supplementary material for: Vitamin B12 deficiency in metformin-treated adults with type 2 diabetes mellitus in Africa: A systematic review and meta-analysis
Source: Metabol Open. 2026 Nov 2;32:100524. doi: 10.1016/j.metop.2026.100524 (PMC13760081; doi:10.1016/j.metop.2026.100524)
Supplement: Supplementary file 3 [file mmc3.docx]

**S3 file. Quality assessment scores of the included studies**

**Table S3.1.** Quality assessment of analytical cross-sectional studies (n = 14)

| **Study** | **Quality assessment tool** | **Item scores** |
| --- | --- | --- |
| Ahmed MA, et.al (2016) | JBI Checklist | Yes, Yes, Yes, Yes, Yes, Yes, Yes, Yes. |
| Sakyi, S. A., et al. (2021) | JBI Checklist | Yes, Yes, Yes, Yes, Yes, Yes, Yes, Yes. |
| Akinlade, K. S., et al. (2015) | JBI Checklist | Yes, Yes, Yes, Yes, No, Yes, Yes, Yes. |
| Wale Tesega, W., et al. (2021) | JBI Checklist | Yes, Yes, Yes, Yes, Yes, Yes, Yes, Yes. |
| Rojbi, I., et al. (2023) | JBI Checklist | Yes, Yes, Yes, Yes, Yes, Yes, Yes, Yes. |
| Ali, H. O., et al. (2026) | JBI Checklist | Yes, Yes, Yes, Yes, Yes, Yes, Yes, Yes. |
| Khan, Z. D. (2023) | JBI Checklist | Yes, Yes, Yes, Yes, Yes, Yes, Yes, Yes. |
| Osama, H., et al. (2016) | JBI Checklist | Yes, Yes, Yes, Yes, No, No, Yes, Yes. |
| Fakkar, N. F. H., et al. (2022) | JBI Checklist | Yes, Yes, Yes, Yes, Yes, No, Yes, Yes. |
| Elsayed, A. M., et al. (2022) | JBI Checklist | Yes, Yes, Yes, Yes, Yes, No, Yes, Yes. |
| Elsaier, A., et al. (2017) | JBI Checklist | Yes, Yes, Yes, Yes, No, No, Yes, Yes. |
| Yakubu, M., et al. (2019) | JBI Checklist | Yes, Yes, Yes, Yes, Yes, Yes, Yes, Yes. |
| Assoudani et al. (2026) | JBI Checklist | Yes, Yes, Unclear, Unclear, Yes, Unclear, Yes, Unclear. |
| Sulaiman et al. (2023) | JBI Checklist | Yes, Yes, Unclear, Yes, Yes, No, Yes, Unclear. |

**The 8 JBI questions for analytical cross-sectional studies.**

**Q1: Were the criteria for inclusion in the sample clearly defined?**

**Q2: Were the study subjects and the setting described in detail?**

**Q3: Was the exposure measured in a valid and reliable way?**

**Q4: Were objective, standard criteria used for measurement of the condition?**

**Q5: Were confounding factors identified?**

**Q6: Were strategies to deal with confounding factors stated?**

**Q7: Were the outcomes measured in a valid and reliable way?**

**Q8: Was appropriate statistical analysis used?**

**Table S3.2. Quality assessment of case-control studies (n = 2)**

| **Study** | **Quality assessment tool** | **Item scores** |
| --- | --- | --- |
| Owhin, S. O., et al. (2019) | JBI Checklist | Yes, Yes, Yes, Yes, Yes, Yes, No, Yes, Yes, Yes |
| Mohammed, A. O. and A. A. Abd Elkarim (2021) | JBI Checklist | Yes, No, Yes, Yes, Yes, Yes, No, Yes, No, Yes |

**The 10 JBI questions for analytical case-control studies.**

**Q1: Were the groups comparable other than the presence of disease (the outcome)?**

**Q2: Were cases and controls matched appropriately?**

**Q3: Were the same criteria used for identification of cases and controls?**

**Q4: Was exposure measured in a standard, valid and reliable way?**

**Q5: Was the exposure period of interest long enough to be meaningful?**

**Q6: Were confounding factors identified?**

**Q7: Were strategies to deal with confounding factors stated?**

**Q8: Were the outcomes of cases and controls measured in the same way?**

**Q9: Was exposure measured in the same way for cases and controls?**

**Q10: Was appropriate statistical analysis used?**

**Table S3.3.** Quality assessment of retrospective cohort studies (n = 3)

| **Study** | **Quality assessment tool** | **Item scores** |
| --- | --- | --- |
| Kondlo, B., et al. (2024) | JBI Checklist | Yes, Yes, Yes, Yes, Yes, No, Yes, Yes, Yes, No, Yes. |
| Mahmoud, S. H., et al. (2025) | JBI Checklist | Yes, Yes, Yes, Yes, Yes, No, Yes, No, No, Yes, Yes. |
| Naidoo et al. (2024) | JBI Checklist | Yes, Yes, Yes, Yes, Unclear, No, Yes, Unclear, No, No, Yes. |

**The 11 JBI questions for retrospective cohort studies (n=2)**

Q1. Were the two groups similar and recruited from the same population?

Q2. Were the exposures measured similarly to assign people to both exposed and unexposed groups?

Q3. Was the exposure measured in a valid and reliable way?

Q4. Were confounding factors identified?

Q5. Were strategies to deal with confounding factors stated?

Q6. Were the participants free of the outcome at the start of the study (or at the moment of exposure)?

Q7. Were the outcomes measured in a valid and reliable way?

Q8. Was the follow-up time reported and sufficient to be long enough for outcomes to occur?

Q9. Was follow-up complete, and if not, were the reasons for loss to follow-up described and explored?

Q10. Were strategies to address incomplete follow-up utilized?

Q11. Was appropriate statistical analysis used?
