# Supplementary material for: Vitamin B12 deficiency in metformin-treated adults with type 2 diabetes mellitus in Africa: A systematic review and meta-analysis
Source: Metabol Open. 2026 Nov 2;32:100524. doi: 10.1016/j.metop.2026.100524 (PMC13760081; doi:10.1016/j.metop.2026.100524)
Supplement: Supplementary file 4 [file mmc5.docx]

**Supplementary Table (SF5). Study-specific diagnostic thresholds for vitamin B12 deficiency, standardized values, and classification for threshold-restricted sensitivity analysis**

| **No.** | **Study (year)** | **Original reported threshold** | **Standardized threshold (pg/mL)** | **Classification** |
| --- | --- | --- | --- | --- |
| 1 | Naidoo et al. (2024) | <148 pmol/L | <200.60 | Included |
| 2 | Fakkar et al. (2022) | <200 pg/mL | <200.00 | Included |
| 3 | Wale Tesega et al. (2021) | <197 pg/mL | <197.00 | Included |
| 4 | Rojbi et al. (2023) | <200 pg/mL | <200.00 | Included |
| 5 | Elsaier et al. (2017) | <200 pg/mL | <200.00 | Included |
| 6 | Akinlade et al. (2015) | <200 pg/dL | <2.00 | Excluded—unit requires verification |
| 7 | Elsayed et al. (2022) | <200 pg/mL | <200.00 | Included |
| 8 | Ali et al. (2026) | <150 pg/mL | <150.00 | Excluded—lower threshold |
| 9 | Khan (2023) | <220 pmol/L | <298.18 | Excluded—higher threshold |
| 10 | Yakubu et al. (2019) | <100 pg/mL + MMA ≥0.4 µmol/L | ----- | Excluded—combined indicators |
| 11 | Kondlo et al. (2024) | ≤200 pmol/L | ≤271.08 | Excluded—higher threshold |
| 12 | Assoudani et al. (2026) | <200 pg/mL | <200.00 | Included |
| 13 | Mohammed & Abd Elkarim (2021) | NR | NR | Excluded—threshold not reported |
| 14 | Sulaiman et al. (2023) | <200 pg/mL | <200.00 | Included |
| 15 | Mahmoud et al. (2025) | <200 pg/mL | <200.00 | Included |
| 16 | Ahmed et al. (2016) | <150 pmol/L | <203.31 | Included |
| 17 | Sakyi et al. (2021) | 4cB12 (ω < −0.5) | ----- | Excluded—alternative indicator |
| 18 | Owhin et al. (2019) | <148 pmol/L | <200.60 | Included |
| 19 | Osama et al. (2016) | <180 pg/mL | <180.00 | Excluded—lower threshold |

**Abbreviations:** MMA, methylmalonic acid; NR, not reported; 4cB12, combined vitamin B12 indicator.

**Notes:** Serum vitamin B12 thresholds were standardized to pg/mL using the conversion factors 1 pg/mL = 0.7378 pmol/L and 1 pg/dL = 0.01 pg/mL. Eleven studies reporting serum vitamin B12 thresholds between 197.00 and 203.31 pg/mL were classified as approximately comparable to the commonly used threshold of 200 pg/mL. This range was defined operationally for the threshold-restricted sensitivity analysis and should not be interpreted as demonstrating identical diagnostic performance across assays. Studies using thresholds outside this range, combined or alternative diagnostic indicators, or unreported thresholds were excluded. Original inequality signs were preserved in the standardized thresholds.
