# Supplementary material for: Vitamin B12 deficiency in metformin-treated adults with type 2 diabetes mellitus in Africa: A systematic review and meta-analysis
Source: Metabol Open. 2026 Nov 2;32:100524. doi: 10.1016/j.metop.2026.100524 (PMC13760081; doi:10.1016/j.metop.2026.100524)
Supplement: Supplementary file 5 [file mmc6.docx]

Supplementary Table (SF6). Coding schemes, study counts, and meta-regression coefficients for categorical moderators included in the exploratory meta-regression.

| **Moderator / Category** | **Code** | **Studies (n)** | **β (95% CI)** | **SE** | **P-value** |
| --- | --- | --- | --- | --- | --- |
| **Geographic region** |  |  |  |  |  |
| East Africa (Reference) | 1 | 4 | Reference | — | — |
| North Africa | 2 | 8 | 0.399 (−0.898 to 1.697) | 0.662 | 0.546 |
| South Africa | 3 | 3 | −0.045 (−1.650 to 1.561) | 0.819 | 0.957 |
| West Africa | 4 | 4 | 0.932 (−0.546 to 2.409) | 0.754 | 0.216 |
| **Laboratory assay** |  |  |  |  |  |
| CLIA (Reference) | 1 | 5 | Reference | — | — |
| ECLIA | 2 | 5 | −0.077 (−1.282 to 1.129) | 0.615 | 0.901 |
| ECLIA + ELISA | 3 | 1 | 1.720 (−0.264 to 3.704) | 1.012 | 0.089 |
| ELISA | 4 | 4 | 1.305 (0.042 to 2.568) | 0.644 | 0.043 |
| HPLC | 5 | 1 | −0.254 (−2.365 to 1.858) | 1.077 | 0.814 |
| Not reported (NR) | 6 | 3 | 0.019 (−1.339 to 1.378) | 0.693 | 0.978 |

**Note**: β represents the meta-regression coefficient on the logit-prevalence scale relative to the reference category. Separate univariable random-effects models were fitted using restricted maximum likelihood (REML) with conventional Wald-based inference, consistent with Table 3. Each model included 19 studies. Analyses were exploratory, and estimates from categories containing only one study should be interpreted cautiously.
