# Supplementary material for: Vitamin B12 deficiency in metformin-treated adults with type 2 diabetes mellitus in Africa: A systematic review and meta-analysis
Source: Metabol Open. 2026 Nov 2;32:100524. doi: 10.1016/j.metop.2026.100524 (PMC13760081; doi:10.1016/j.metop.2026.100524)
Supplement: Supplementary file 6 [file mmc7.docx]

**Table 1. Reported determinants of vitamin B12 deficiency among metformin-treated adults with type 2 diabetes mellitus in Africa**

| **Study** | **Country** | **Determinant/exposure** | **Exposure definition** | **Adjusted effect estimate** | **95% CI** | **Direction of association** |
| --- | --- | --- | --- | --- | --- | --- |
| Ahmed MA et al., 2016 | South Africa | Long duration of metformin use | Longer duration of metformin use | AOR = 2.68 | 1.03–6.97 | Positive |
| Kondlo B et al., 2024 | South Africa | Older age | Older age | AOR = 6.67 | 1.16–38.30 | Positive |
| Kondlo B et al., 2024 | South Africa | Coloured race | Coloured race | AOR = 7.78 | 1.78–34.20 | Positive |
| Ali HO et al., 2026 | Uganda | High metformin dose | High metformin dose | AOR = 4.634 | 1.124–19.105 | Positive |
| Ali HO et al., 2026 | Uganda | Long duration of metformin use | Longer duration of metformin use | AOR = 4.044 | 1.441–11.353 | Positive |
| Ali HO et al., 2026 | Uganda | Poor glycemic control | High HbA1c | AOR = 2.448 | 1.041–6.238 | Positive |
| Yakubu M et al., 2019 | Ghana | Very high body fat | Very high body-fat percentage | AOR = 2.98 | 1.47–6.05 | Positive |
| Yakubu M et al., 2019 | Ghana | Metformin duration 5–9 years | 5–9 years of metformin use | AOR = 2.83 | 1.03–7.81 | Positive |
| Yakubu M et al., 2019 | Ghana | Long duration of metformin use | Longer duration of metformin use | AOR = 4.17 | 1.41–12.33 | Positive |
| Muhammad F, Enikuomehin A, 2023 | Nigeria | High metformin dose | High metformin dose | AOR = 3.50 | 1.33–9.368 | Positive |

**Abbreviations:** AOR, adjusted odds ratio; CI, confidence interval; HbA1c, glycated hemoglobin.

**Note:** Effect estimates are presented as reported by the individual studies. Because determinants, exposure definitions, and reference categories adjusted estimates were not pooled quantitatively. “Positive” indicates that the reported exposure was associated with higher odds of vitamin B12 deficiency.
